# Ischemic arterial events and atherosclerosis in patients with systemic sclerosis: a population-based case-control study

**DOI:** 10.1186/ar4267

**Published:** 2013-08-14

**Authors:** Annica Nordin, Kerstin Jensen-Urstad, Lena Björnådal, Susanne Pettersson, Anders Larsson, Elisabet Svenungsson

**Affiliations:** 1 Rheumatology Unit, Department of Medicine, Karolinska University Hospital, Solna, Karolinska Institutet, SE-171 76 Stockholm, Sweden; 2 Department of Clinical Physiology, Södersjukhuset, Karolinska Institutet, SE-171 76 Stockholm, Sweden; 3 Division of Nursing, Department of Neurobiology, Care Sciences and Society, Karolinska Institutet, SE-171 76 Stockholm, Sweden; 4 Department of Clinical Chemistry and Pharmacology, Akademiska Hospital, SE-751 85 Uppsala, Sweden

## Abstract

**Introduction:**

While microvascular disease is well described in systemic sclerosis (SSc), it is still unclear whether the occurrence of ischemic macrovascular events and atherosclerosis is enhanced among patients with SSc.

**Methods:**

In this study, 111 SSc patients (74% of prevalent cases in Stockholm County) and 105 age- and sex-comparable population controls were investigated. Previous ischemic arterial events were tabulated. As surrogate measures of atherosclerosis, plaque occurrence and intima-media thickness (IMT) were determined with carotid ultrasound and the ankle-brachial index (ABI) was calculated. Traditional cardiovascular risk factors were recorded and we also measured biomarkers indicating systemic inflammation and endothelial activation/dysfunction.

**Results:**

Mean age was 62 ± 12 years for patients and controls. Ischemic arterial events were more common, due to increased occurrence of ischemic heart disease (IHD) and ischemic peripheral vascular disease (IPVD), in the patient group (12% vs. 4%, *P *= 0.03 and 9% vs. 0%, *P *= 0.003 respectively). On a group level, there was no difference regarding the occurrence of ischemic cerebrovascular disease, the frequency of plaques, IMT or ABI between SSc patients and controls. Subgroup analyses revealed that patients with anticentromere antibodies (ACA+) had more plaques and more ischemic arterial events compared to other SSc patients (67% vs. 39% and 32% vs. 11%; *P *= 0.006 and *P *= 0.01, respectively) and compared to controls (67% vs. 41% and 32% vs. 7%, *P *= 0.02 and *P *= 0.0003, respectively). Biomarkers of inflammation/endothelial activation were generally increased among SSc patients.

**Conclusions:**

Patients with SSc are at enhanced risk for IHD and IPVD. The ACA+ SSc subgroup was particularly affected with both ischemic arterial events and premature atherosclerosis. The microvascular vulnerability of ACA+ patients is previously well documented. We demonstrate that ACA+ SSc patients have an enhanced risk of macrovascular injury as well. This group should be followed closely and modifiable cardiovascular risk factors should be treated at an early stage.

## Introduction

Systemic sclerosis (SSc) is an autoimmune rheumatic disease characterized by fibrosis of the skin, microvasculopathy and involvement of internal organs. Patients with SSc have a shortened lifespan mainly due to heart and lung manifestations [1]. In other autoimmune diseases such as systemic lupus erythematosus (SLE) and rheumatoid arthritis (RA) the occurrence of premature cardiovascular morbidity and subclinical atherosclerosis has been well documented [2,3]. Whether ischemic macrovascular disease and/or accelerated atherosclerosis also are features of SSc is unclear. Most previous studies are fairly small and results contradictive [4-6], however, a recent meta-analysis concluded that carotid intima-media thickness (IMT) was greater in SSc patients than in controls [7].

The pathogenesis of SSc is unknown, but activation of B cells results in the production of antinuclear antibodies (ANA) in 90% of patients. The ANA specificities most commonly demonstrated in SSc; anticentromere (ACA) and antitopoisomerase 1 (ATA) antibodies, characterize two different clinical SSc subsets. In limited cutaneous scleroderma (lcSSc, 80% of SSc) the occurrence of ACA is common [8] and Raynaud's phenomena may precede fibrotic skin by several years [1]. Diffuse cutaneous systemic sclerosis (dcSSc) is less common, associated with ATA, and an extensive skin involvement and pulmonary fibrosis often parallel or may even precede Raynaud's phenomena.

In this study, we investigated the prevalence of ischemic arterial events and measures of atherosclerosis in patients with SSc and in controls from the general population. Traditional cardiovascular risk factors were tabulated. Since both atherosclerosis and cardiovascular disease are known to be associated with systemic inflammation, we also investigated the distribution of a large set of inflammatory and endothelial biomarkers.

## Methods

### Patients and controls

All participants were >18 years old and recruited from the adult population in Stockholm County (*N *= 1,534,272) between August 2006 and December 2009. Prevalent cases with SSc were identified at the three Rheumatology clinics associated with general/university hospitals and from all rheumatologists with private practice. All dermatologists, gastroenterologists, specialists in respiratory medicine, hand surgeons and general practitioners were contacted twice and asked to inform us about their patients with SSc. We identified 149 cases fulfilling the American College of Rheumatology (ACR) criteria for SSc [9], which corresponds to a prevalence of 97 adult SSc cases/million. A total of 74% participated in the study. According to our review of medical records, the remaining 26% did not differ from the included patients regarding age, disease duration or severe organ manifestations. Control subjects were recruited from the same population through use of the national registration number, which includes date of birth and is coded for sex. The sex-matched person with the birth date closest to the patient was contacted and asked to participate. Some 65% of these 'first choice' controls accepted to participate. When the 'first choice' declined, the second-closest person was asked, until a control subject gave his/her consent. A diagnosis of SSc was the only exclusion criteria. A total of 105 controls were included.

The local ethics committee of the Karolinska University Hospital approved the study and all participants gave written informed consent.

### Study protocol

All participants underwent a structured interview and a physical examination by a rheumatologist (AN, LB).

The patients were classified as lcSSc or dcSSc using the LeRoy classification [10]. Skin score was measured with the modified Rodnan skin score [11] and disease activity with the preliminary European Scleroderma Study Group (EScSG) activity index [12]. The Medsger scale of disease damage was used, with the exception that the joint/tendon and muscular item was not performed [13]. Organ involvement was defined as: suspected pulmonary hypertension (sPH): tricuspidalis V-max >2.9m/s on Doppler ultrasound. Pulmonary fibrosis: signs of fibrosis on X-ray or high-resolution computed tomography (HRCT). HRCT was performed in 90% of the patients. Myositis: muscular weakness and elevated creatine kinase and signs of inflammation on magnetic resonance imaging (MRI), electromyography (EMG) or muscular biopsy. Arthritis: swollen joints on examination or arthritis documented in the journals. Kidney involvement: a history of scleroderma renal crisis or >1 on the Medsger scale for kidney damage [13].

Ischemic arterial events were defined as:

1. Ischemic heart disease (IHD): myocardial infarction (confirmed by electrocardiography and a reversible rise in plasma creatine kinase, muscle and brain fraction (CK-MB) or troponin T) or angina pectoris (confirmed by an exercise stress test).

2. Ischemic cerebrovascular disease (ICVD): cerebral infarction (confirmed by computed tomography) or transitory ischemic attacks (TIA, defined as transient focal symptoms from the brain or retina with a maximum duration of 24 hours).

3. Ischemic peripheral vascular disease (IPVD): intermittent claudication + ankle-brachial index (ABI) <0.9 or peripheral arterial thrombosis/embolus (confirmed by angiogram or Doppler flow studies).

4. Any ischemic arterial event that includes 1 to 3 above

### Measures of atherosclerosis

The left and right common carotid arteries and bifurcation areas were scanned for presence of plaque and images for IMT measurements were received using a duplex scanner (Siemens Acuson Sequoia, Mountain View, CA, USA) with a 7.0 MHz linear array transducer. Scans were digitalized for offline analysis. The subject's head was tilted to get the picture of the common carotid artery (CCA) just proximal to the bulb placed horizontally across the screen. Pictures were frozen synchronously with the R wave on the electrocardiogram. The IMT was defined as the distance between the leading edges of the luminal echo and the media/adventitia echo [14]. The IMT was calculated as the intima-media area divided by the measured length (10mm) on one scan. Plaques were defined as a local increase in wall thickness of >1 mm and 100% increase in wall thickness compared to the adjacent wall. A technician recorded all measurements and a single experienced reader (KJU) interpreted the registrations without knowledge of patient/control status or other test results.

To obtain the ABI, the patients were placed in a supine position. The highest systolic pressure of the tibialis posterior or dorsalis pedis for each foot were divided by the highest brachial systolic pressure to obtain an ankle-brachial pressure ratio.

### Laboratory analyses

Antinuclear antibodies (ANA) were analyzed by immunofluorescence (IFL) on sections of rat liver and HEp-2 cells (Immunoconcepts, Sacramento, CA, USA). Anticentromere antibodies (ACA) and antitopoisomerase 1 antibodies (ATA) were analyzed by the BioPlex 2200 ANA screen system (Bio-Rad, Hercules, CA, USA). High-sensitivity C-reactive protein (hsCRP), α-1 antitrypsin, orosomucoid and fibrinogen were measured using BN ProSpec System (Dade Behring, Deerfield, IL, USA). Intercellular adhesion molecule 1(ICAM-1), vascular cell adhesion molecule 1(VCAM-1), interleukin 6 (IL-6) and vascular endothelial growth factor (VEGF) were analyzed by sandwich ELISA kits (DY720, DY206, DY137 and DY293B, R & D Systems, Minneapolis, MN, USA). Cutoff values were 15 pg/mL for ICAM-1, VCAM-1 and VEGF and 6 pg/mL for IL-6.

Cystatin C (reagent: 1014, Gentian, Moss, Norway) was analyzed on an Architect Ci8200^™ ^analyzer (Abbott, Abbot Park, IL, USA). The total analytical imprecision of the cystatin C method was 1.1% at 1.25 mg/L and 1.4% at 5.45 mg/L. The equation used for calculating glomerular filtration rate (GFR) in mL/min/1.73 m^2 ^from the cystatin C results in mg/mL was y = 79.901x^-1.4389^[15].

von Willebrand factor (vWF) was measured by sandwich ELISA (Dako, Glostrup, Denmark) [16].

### Statistical methods

Characteristics were summarized for all patients and controls. Skewed continuous variables were log transformed to obtain a normal distribution, if possible. Groups were compared using ANOVA, Mann-Whitney *U *test and X^2 ^test as needed. Odds ratio (OR) and 95% confidence intervals (CI) were calculated from 2 × 2 contingency tables or from nominal logistic regression models. For continuous variables, we used standard least squares linear regression to calculate standardized regression coefficients (β) and *P *values. Variables were sorted into functional groups (traditional risk factors, and so on. see Table 1). After age adjustment, the variables within each group, which were representative and most significantly, as determined by lowest *P *value, associated with plaque occurrence were entered into a multivariable-adjusted model. Due to limited number of plaques, we restricted the number of variables to six. Calculations were done using JMP software (SAS Institute, Cary, NC, USA). A *P *value < 0.05 was considered statistically significant.

**Table 1:** Characteristics of systemic sclerosis patients and controls at inclusion.

	Patients (*n *= 111)	Controls (*n *= 105)	*P *value
Age, years	61.8 ± 12.5	61.5 ± 12.3	ns
Gender female % (n)	81 (90)	86 (90)	ns
**Disease characteristics**			
Disease duration (years)	9.4 (5.6-17.4)	-	-
Diffuse cutaneous % (n) Limited cutaneous % (n)	22 (24) 78 (87)	- -	-
Pulmonary fibrosis % (n)	46 (51)	-	-
Suspected pulmonary hypertension % (n)	15 (17)	0	-
Raynaud % (n)	95 (105)	11 (12)	-
Digital ulcers ever % (n)	39 (43)	0	-
Calcinosis % (n)	27 (30)	0	-
Myositis % (n)	9 (10)	0	-
Arthritis % (n)	29 (32)	4 (4)	-
Kidney involvement % (n)	7 (8)	0	-
Skin score	6 (3-10)	0	-
Disease activity	0.5 (0-2)	-	-
Disease severity	5 (3-6)	-	-
**Autoantibodies** Anticentromere (ACA) % (n) Antitopoisomerase 1 (ATA) % (n) Antinuclear (ANA) neg % (n)	32 (35) 23 (25) 7 (8)	- - -	
**Traditional cardiovascular risk factors**			
Body mass index (kg/m^2^)	24.2 ± 3.80	26.0 ± 3.7	0.0005
Waist-hip ratio	0.83 ± 0.09	0.84 ± 0.08	ns
Ever smoked % (n) Current smoker % (n)	53 (59) 11 (12)	44 (46) 7 (7)	ns ns
Systolic BP (mmHg) Diastolic BP (mmHg) Hypertension % (n)	123.18 ± 17.94 71.63 ± 10.65 30 (33)	124.09 ± 18.59 74.5 ± 9.49 20 (21)	ns 0.04 ns
P-glucose (mmol/l) Diabetes % (n)	5.45 ± 1.17 6 (7)	5.43 ± 1.34 7 (7)	ns ns
Cholesterol (mmol/l) LDL (mmol/l) HDL (mmol/l) Triglycerides (mmol/l)	5.20 ± 1.04 3.32 ± 0.93 1.3 (1.0-1.5) 0.99 (0.76-1.5)	5.24 ± 1.15 3.37 ± 0.91 1.4 (1.2-1.7) 0.93 (0.67-1.3)	ns ns ns (0.09) 0.03
**Inflammatory biomarkers **			
hsCRP (mg/l)	2.2 (1.0-4.3)	1.75 (0.76-3.2)	0.05
Orosomucoid (g/l)	0.86 ± 0.23	0.77 ± 0.17	0.0009
Alpha1antitrypsine (g/l)	1.5 (1.4-1.6)	1.4 (1.2-1.5)	<0.0001
Fibrinogen (g/l)	3.77 (3.3-4.34)	3.53 (2.80-4.25)	0.03
Sedimentation rate (mm) Interleukin-6 (pg/ml)	14 (9-26) 29.9 (21.4-40.9)	10 (7-16.5) 27.1 (21.2-32.5)	0.002 ns (0.08)
**Endothelial biomarkers**			
VCAM-1 (ng/l)	698 (546-807)	531 (431-719)	<0.0001
ICAM-1 (ng/l)	376 (314-472)	330 (254-450)	0.002
VEGF (pg/ml)	182 (143-258)	156 (103-239)	0.009
vWF (IU/ml)	1.25 (0.90-1.70)	0.85 (0.58-1.36)	<0.0001
**Renal function **			
eGFR (mL/min/1.73 m^2^)	74 (50-116)	104 (89-126)	0.01

Distributions are given as % (n), median (interquartile range) or mean ± standard deviation. BP, blood pressure; LDL, low-density lipoprotein; HDL, high-density lipoprotein; hsCRP, high-sensitivity C reactive protein; VCAM-1, vascular cell adhesion molecule 1; ICAM-1, intercellular adhesion molecule 1; VEGF, vascular endothelial growth factor; vWF, von Willebrand factor; eGFR, estimated glomerular filtration rate, based on cystatin C measurements.

## Results

### Basic characteristics

At inclusion patients with SSc had lower body mass index, lower diastolic blood pressure, but higher triglycerides and biomarkers of systemic inflammation/endothelial activation as compared to controls. Baseline characteristics and comparisons between patients and controls are given in Table 1.

### Occurrence of ischemic arterial events and measures of atherosclerosis

Ischemic arterial events were more common in patients than in controls (Table 2), due to more prevalent IHD and IPVD, while ICVD did not differ between groups (events are tabulated in detail, see Additional file 1,Table 1). Of participants with IHD, seven patients and three controls were diagnosed with myocardial infarction. The remaining IHD subjects had angina pectoris verified with an exercise test, of these two patients and one control had undergone an angiogram, all with normal cardiac vessels. Five patients and one control had had multiple events.

**Table 2:** Ischemic arterial events and surrogate measures of atherosclerosis in systemic sclerosis patients versus controls.

	Patients (*n *= 111)	Controls (*n *= 105)	Odds ratio (95% CI)	*P *value
**Any arterial event **% (n)	18 (20)	7 (7)	**3.1 (1.2-7.6)**	**0.01**
**IHD **% (n)	12 (13)	4 (4)	**3.3 (1.1-10.6)**	**0.03**
**ICVD **% (n)	3 (4)	3 (3)	0.9 (0.2-4.7)	ns
**IPVD **% (n)	8 (9)	1 (1)	**9.2 (1.1-73.7)**	**0.02**
**Plaques **% (n)	48 (52)	41 (43)	1.3 (0.7-2.3)	ns
			**β-coefficient**	
**IMT **mm	0.68 ± 0.13	0.68 ± 0.13	-0.02	ns
**ABI**	1.13 (1.1-1.2)	1.12 (1.1-1.2)	-0.08	ns

Any arterial event refers to any ischemic arterial event, including IHD, ICVD and IPVD. IHD, ischemic heart disease; ICVD, ischemic cerebrovascular disease; IPVD, ischemic peripheral vascular disease; IMT, intima-media thickness; ABI, ankle-brachial index.

The majority of the IPVD patients (78%) in our study had severe macrovasculopathy requiring surgical intervention. One SSc patient and the only control had IPVD based on ABI <0.9 together with typical symptoms of claudication (see Additional file 1, Table 1).

Plaque occurrence, IMT and ABI did not differ between patients and controls (Table 2). Six patients and two controls had an ABI below 0.9 and one patient and one control had an ABI higher than 1.4.

### Risk factors for ischemic arterial events

As expected, all ischemic arterial events were positively associated with age. We therefore adjusted all further calculations for age. All variables presented in Table 1 were investigated for associations with ischemic arterial events. All variables, which after age adjustment remained associated with at least one of the respective cardiovascular outcomes (any event, IHD or IPVD), are presented in Table 3. Age, kidney involvement, cystatin C estimated (e)GFR were associated with any ischemic arterial event and with IHD. ACA+ and skin score were associated with any ischemic arterial event and IPVD. Interestingly, patients with ATA had less total events and less IHD. IHD was also associated with endothelial biomarkers. Due to few events, we were not able to perform further multivariable calculations.

**Table 3:** Variables associated with ischemic arterial events in systemic sclerosis patients after age adjustment.

	Any event (*n *= 20)		IHD (*n *= 13)		IPVD (*n *= 9)	
	**Odds ratio (95% CI)**	***P *value**	**Odds ratio (95% CI)**	***P *value**	**Odds ratio (95% CI)**	***P *value**

**Age**	**1.10 (1.05-1.17)**	**<0.0001**	**1.06 (1.01-1.13)**	**0.02**	**1.13 (1.05-1.26)**	**0.0005**

**Associations after adjustment for age**						

Kidney disease	**8.8 (1.5-55.8)**	**0.01**	**6.6 (1.12-36.15)**	**0.04**	ND	0.3
Skin score	**1.13 (1.03-1.24)**	**0.008**	1.07 (0.97-1.18)	0.1	**1.16 (1.02-1.32)**	**0.02**
Disease severity	**1.29 (1.05-1.62)**	**0.02**	1.11 (0.88-1.39)	0.4	1.15 (0.85-1.55)	0.3
ACA+	**3.61 (1.21-11.3)**	**0.02**	1.9 (0.55-6.45)	0.2	6.8 (1.37-45.3)	**0.02**
ATA+	0.18 (0.01-1.01)	0.05	**0.00003 (0-0.6)**	**0.01**	0.83 (0.04-5.67)	0.9
VCAM-1	5.30 (0.51-62.1)	0.2	**27.3 (2.52-427. 4)**	**0.005**	0.37 (0.02-5.46)	0.2
VEGF	0.68 (0.24-1.84)	0.4	**0.29 (0.09-0.93)**	**0.03**	0.52 (0.12-2.33)	0.4
eGFR	**0.26 (0.10-0.62)**	**0.003**	**0.32 (0.12-0.81)**	**0.02**	0.57 (0.15-3.65)	0.5

IHD, ischemic heart disease; IPVD, ischemic peripheral vascular disease; CI, confidence interval; ACA, anticentromere antibody; +, positivity; ATA, antitopoisomerase 1 antibody; VCAM-1, vascular cell adhesion molecule 1; VEGF, vascular endothelial growth factor; eGFR, estimated glomerular filtration rate, based on cystatin C measurements.

### Risk factors for atherosclerosis

The investigated measures of atherosclerosis were all positively associated with age. Thus, all further calculations were age-adjusted. Each variable presented in Table 1 was investigated for association with plaque occurrence, IMT and ABI. All variables, which after age adjustment remained associated with at least one of the respective atherosclerosis outcomes (plaque, IMT or ABI), are presented in Table 4.

**Table 4:** Variables associated with measures of atherosclerosis in systemic sclerosis patients after age adjustment.

	Plaques (*n *= 52)		IMT		ABI	
	**Odds ratio** **(95% CI)**	***P *value**	**β-coefficient**	***P *value**	**β-coefficient**	***P *value**

**Age**	**1.09 (1.05-1.14)**	**<0.0001**	**0.66**	**<0.0001**	**-0.20**	**0.03**

**Associations after adjustment for age**						

Gender female	0.39 (0.12-1.18)	0.1	**-0.19**	**0.0001**	-0.12	0.2
ACA+	**2.9 (1.2-7.5)**	**0.02**	-0.01	0.9	-0.14	0.2
Digital ulcers	**2.6 (1.1-6.6)**	**0.03**	0.10	0.2	-0.09	0.4
Kidney disease	**11.5 (1.7-232)**	**0.03**	-0.05	0.5	0.05	0.6
Disease activity	**1.6 (1.04-2.5)**	**0.03**	0.10	0.2	**-0.34**	**0.0004**
Disease severity	**1.3 (1.1-1.5)**	**0.005**	0.12	0.1	0.13	0.2
Waist-hip ratio	2.6 (0.01-645)	0.7	0.09	0.2	**0.19**	**0.04**
Ever smoked	**2.5 (1.05-6.7)**	**0.04**	0.10	0.2	**0.24**	**0.01**
Systolic BP	**1.0 (1.0-1.1)**	**0.02**	**0.28**	**0.0008**	-0.06	0.6
Diastolic BP	**1.1 (1.0-1.1)**	**0.008**	0.13	0.1	-0.02	0.9
Interleukin-6	**3.5 (1.5-9.3)**	**0.003**	0.12	0.1	-0.13	0.2
VCAM-1	**9.4 (1.8-58.3)**	**0.008**	-0.05	0.5	0.03	0.8
ICAM-1	0.32 (0.05-1.98)	0.2	**0.16**	**0.04**	-0.12	0.2
eGFR	**0.4 (0.2-0.99)**	**0.04**	0.11	0.2	-0.04	0.7

IMT, intima-media thickness; ABI, ankle-brachial index; CI, confidence interval; ACA, anticentromere antibodies; +, positivity; BP, blood pressure; VCAM-1, vascular cell adhesion molecule 1; ICAM-1, intercellular adhesion molecule 1; eGFR, estimated glomerular filtration rate, based on cystatin C measurements.

Plaque occurrence was associated with smoking, higher blood pressure, impaired kidney function, high levels of IL-6 and VCAM-1 and with ACA+.

As expected, we noted that IMT was strongly associated with systolic blood pressure and with male gender. ABI was associated with disease activity, waist-hip ratio and smoking.

Several variables were associated with plaque occurrence and we included age and the variables with the lowest *P *values in each 'functional group' in a multivariable-adjusted logistic regression. In this model only ACA+ and high levels of IL-6 remained convincingly associated with plaque occurrence, while the association with diastolic blood pressure was of borderline significance (Table 5).

**Table 5:** Multivariable adjusted model for occurrence of carotid plaques.

	Plaques	
**Functional groups** **and included variables**	**Odds ratio** **(95% CI)**	***P *value**

Age	**1.1 (1.03-1.16)**	**0.003**
**Disease characteristics**		
ACA+	**4.0 (1.26-14.1)**	**0.02**
**Traditional risk factors**		
Diastolic blood pressure (mmHg)	**1.1 (1.01-1.11)**	**0.04**
**Inflammatory biomarkers**		
IL-6 (pg/ml)	**3.8 (1.3-13.3)**	**0.01**
**Endothelial biomarkers**		
VCAM-1 (ng/ml)	1.5 (0.15-14.7)	0.7
**Kidney function test**		
eGFR (mL/min/1.73 m^2^)	0.3 (0.04-1.07)	0.07

CI, confidence interval; ACA, anticentromere antibodies; +, positivity; IL, interleukin; VCAM-1, vascular cell adhesion molecule 1; eGFR, estimated glomerular filtration rate, based on cystatin C measurements.

### Subgroup analysis of ACA+ SSc patients

Finally, due to the observed association between ACA+ and any ischemic arterial event and plaque occurrence, we stratified by ACA status and compared the groups with respect to all variables in Table 1. There were 34 ACA+ patients and 77 ACA-patients. In the ACA+ group disease duration was longer (*P *= 0.04) and dcSSc, pulmonary fibrosis, and ATA antibodies were less common (*P *< 0.05) compared with the ACA-group. Of traditional risk factors, waist-hip ratio, P-glucose and triglycerides levels were lower in ACA+ patients (*P *≤ 0.05). Levels of orosomucoid and ICAM-1 were also lower in the ACA+ group (*P *≤ 0.05) (see Additional file 1, Table 2).

**Figure 1:**
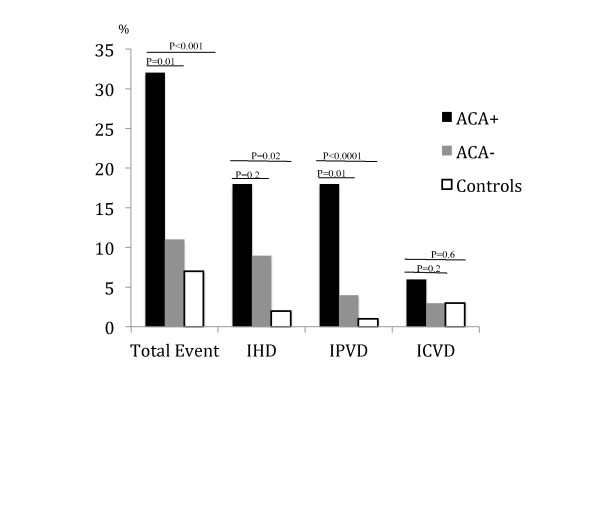
**Ischemic arterial events in ACA+ and ACA-patients and in controls**. Bar graph describing the frequency of ischemic arterial events in systemic sclerosis patients with (+) or without (-) anticentromere antibodies (ACA) and in comparable controls. Ischemic arterial events are defined as: Ischemic heart disease (IHD): myocardial infarction (confirmed by electrocardiography and a reversible rise in plasma creatine kinase, muscle and brain fraction (CK-MB) or troponin T) or angina pectoris (confirmed by exercise stress test). Ischemic cerebrovascular disease (ICVD): cerebral infarction (confirmed by computed tomography) or transitory ischemic attacks (TIA, defined as transient focal symptoms from the brain or retina with a maximum duration of 24 hours). Ischemic peripheral vascular disease (IPVD): intermittent claudication + ABI <0.9 or peripheral arterial thrombosis/embolus (confirmed by angiogram or Doppler flow studies). *P *values between ACA+ versus ACA- are adjusted for age, gender and disease duration and *P *values for ACA+ versus controls are adjusted for age and gender.

**Figure 2:**
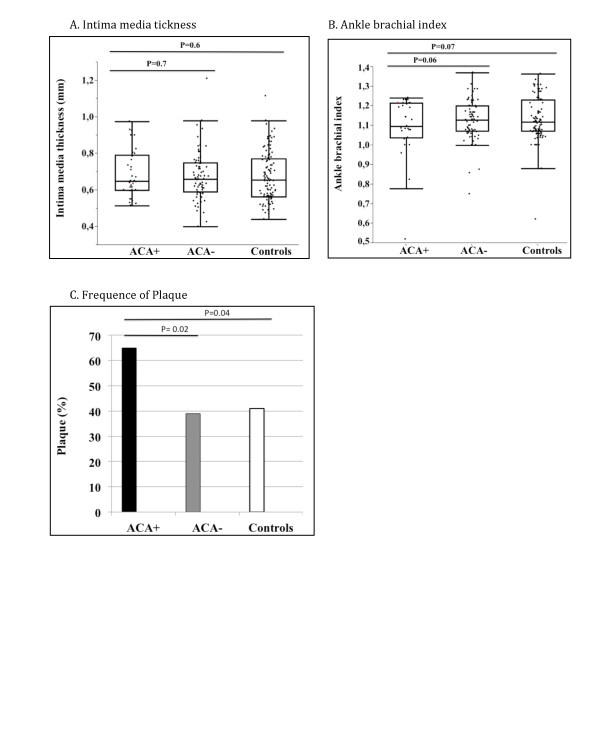
**Measures of atherosclerosis in ACA+ and ACA-patients and in controls**. Outlier box plots of **(A) **intima-media thickness and **(B) **ankle-brachial index in systemic sclerosis patients with (+) or without (-) anticentromere antibodies (ACA) and in comparable controls. The median, interquartile range (IQR) and the lowest value still within 1.5 IQR of the lower quartile, and the highest value still within 1.5 IQR of the upper quartile are represented by the box and whiskers. The dots represent the individual samples. **(C) **is a bar graph describing the frequency of plaques (%) in ACA+, ACA-patients and controls. *P *values between ACA+ versus ACA- are adjusted for age, gender and disease duration and *P *values for ACA+ versus controls are adjusted for age and gender.

**Figure 3:**
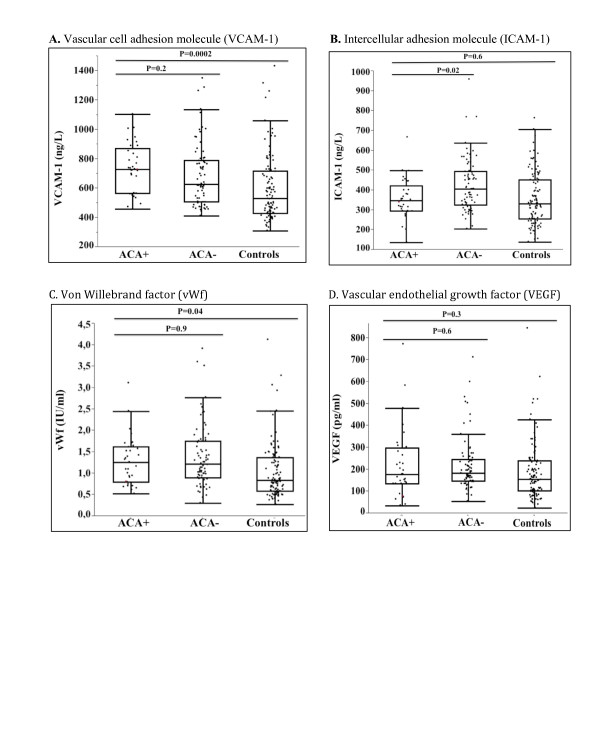
**Markers of endothelial activation in ACA+ and ACA-patients and in controls**. Outlier box plots of **(A) **vascular cell adhesion molecule 1 (VCAM-1), **(B) **intercellular adhesion molecule 1 (ICAM-1), **(C) **von Willebrand factor (vWf) and **(D) **vascular endothelial growth factor (VEGF) in systemic sclerosis patients with (+) or without (-) anticentromere antibodies (ACA) and in comparable controls. The median, interquartile range (IQR) and the lowest value still within 1.5 IQR of the lower quartile, and the highest value still within 1.5 IQR of the upper quartile are represented by the box and whiskers. The dots represent the individual samples. *P *values between ACA+ versus ACA- are adjusted for age, gender and disease duration and *P *values for ACA+ versus controls are adjusted for age and gender.

Occurrence of vascular events, measures of atherosclerosis and levels of endothelial markers in ACA+ and ACA-patients (adjusted for age, sex and disease duration), and in ACA+ patients and controls (adjusted for age and sex) are demonstrated in Table 6, Figure 1, Figure 2 and Figure 3.

**Table 6:** Ischemic arterial events and surrogate measures of atherosclerosis in ACA+ patients versus ACA-patients and controls.

	Occurrence of events and atherosclerosis			ACA+ vs. ACA-		ACA+ vs. controls	
	**ACA+** **(*n *= 34)**	**ACA-** **(*n *= 77)**	**Controls** **(*n *= 105)**	Odds ratio** (95% CI)	*P *value**	Odds ratio* (95% CI)	***P ***value*

Any arterial event % (n)	32 (11)	11 (9)	7 (7)	**5.1 (1.57-18.6) **	**0.01**	**8.5 (2.6-31.7)**	**0.0003**
IHD % (n)	18 (6)	9 (7)	4 (4)	2.6 (0.7-10.0)	0.2	**4.8 (1.2-20.7)**	**0.02**
ICVD % (n)	6 (2)	3 (2)	3 (3)	4.0 (0.4-49.0)	0.2	2.0 (0.2-13.9)	0.6
IPVD % (n)	18 (6)	4 (3)	1 (1)	**9.1 (1.6-79.1)**	**0.01**	**70.1 (6.5-2524)**	**<0.0001**
Plaque % (n)	65 (22)	39 (30)	41 (43)	**3.1(1.2-8.5)**	**0.02**	**2.8 (1.1-7.6)**	**0.04**
				**β-coefficient**		**β-coefficient**	
IMT mm	0.69 ± 0.13	0.68 ± 0.13	0.68 ± 0.13	0.03	0.7	0.01	0.6
ABI	1.1(1.03-1.2)	1.1 (1.17-1.2)	1.1 (1.07-1.2)	-0.12	0.08	-0.14	0.07

Distributions are given as %, median (interquartile range) or mean ± standard deviation. **adjusted for age, sex and disease duration; *adjusted for age and sex. ACA, anticentromere antibodies; +, positivity; **-**, negativity; CI, confidence interval; IHD, ischemic heart disease; ICVD, ischemic cerebrovascular disease; IPVD, ischemic peripheral vascular disease; IMT, intima-media thickness; ABI, ankle-brachial index.

A higher total of ischemic arterial events, IPVD, and more plaques were observed in ACA+ patients as compared to both ACA-patients and controls.

## Discussion

The first major finding in this population-based study is that ischemic arterial events, specifically IHD and IPVD, are more common in patients with SSc, while ICVD and measures of atherosclerosis did not differ on a group level as compared to population controls. Second, the ACA+ SSc subgroup was more affected with ischemic arterial events and with carotid plaques in comparison both to other SSc patients and to controls.

The observed high prevalence of IPVD in patients with SSc highlights an important clinical problem recently also reported by Man *et al. *[17]. Youssef *et al. *[18] found an even higher prevalence of IPVD in females with lcSSc compared with controls (58% vs.10%). More claudication, as a sign of IPVD, was also reported in SSc patients by Veale and collaborators [19].

Despite the higher prevalence of IPVD, SSc patients did not, as a group, have lower ABI than controls. This is in line with three previous studies [20-22], but contrasts with Ho *et al. *[23]. In our study, ACA+ patients had more IPVD compared to both ACA-patients and controls and there was also a trend toward lower ABI in the ACA+ group. Wan *et al*. examined ABI in 119 SSc patients and found no difference between patients with lcSSc and dcSSc, but interestingly ACA+ seemed to prevail among patients with symptomatic ischemic disease [24]. Previous studies have identified ACA+ as a predictor for severe digital loss [25,26]. Thus, in addition to microvascular disease, the large arteries of the extremities seem to contribute significantly to the severe distal ischemic damage seen in many SSc patients, especially in the ACA+ subgroup.

We report a three-fold increased occurrence of IHD in SSc patients and even higher figures in the ACA+ subgroup. This is in line with the recently reported incidence of myocardial infarction [17] and self-reported IHD [27], both of which were two-fold enhanced in SSc patients versus the general population. Older autopsy studies described that myocardial infarctions were common in SSc patients, despite normal coronary arteries [28,29]. Akram *et al*. evaluated 179 angiograms from SSc patients but did not find an increased prevalence of coronary atherosclerosis [30] and normal angiograms were more common than expected among 11 SSc patients with myocardial infarction [31]. These studies suggest that vasospasm rather than atherosclerosis is a major pathogenic mechanism behind SSc-related heart disease. On the other hand, MRI studies have found that calcification of cardiac vessels are common in SSc [32]. The inconsistent results of previous work may partly be explained by our observation that atherosclerotic plaque occurrence is selectively enhanced in the ACA+ subgroup of SSc patients.

In our study, impaired kidney function was highly associated with IHD, a finding that is supported by Derk *et al*., who reported that the majority of SSc patients with IHD had renal failure [30].

Cerebrovascular disease was, in accordance with Youssef *et al*., equally common in SSc patients and controls [18]. However, recently Man *et al*. reported enhanced incidence of stroke in a large epidemiological study [17].

On a group level, there was no difference between patients and controls concerning atherosclerosis, measured as plaques occurrence, IMT or ABI. Our study population was fairly old and the plaque frequency was high in both patients and controls. Previous studies are smaller and the majority measured only IMT. Some report, in agreement with us, similar IMT in patients and controls [5,33-37], others found thicker IMT in SSc patients [20,38-40]. Only three studies examined the frequency of plaques, but their definitions of plaque differ [20,23,41]. We had no exclusion criteria, while some studies excluded SSc patients with manifest cardiovascular disease (CVD) [4,20,35,36]. Furthermore, the selection of comparators is important. The healthy controls in previous studies are likely to have less atherosclerosis than our population-based controls. Methods, definition and selection procedures of both patients and controls are likely to contribute to the heterogeneity of previous studies, reviewed in a recent meta-analysis [7].

Generally, levels of endothelial and inflammatory biomarkers were higher in patients compared to controls, demonstrating that SSc is a low-grade inflammatory disease. Of investigated inflammatory biomarkers, IL-6 remained independently associated with plaques. For SSc this is a new observation but IL-6 has been identified as a risk factor for subclinical coronary atherosclerosis in RA [42], and it has also been linked to poor prognosis in patients with IHD from the general population [43].

VCAM-1 was associated with both IHD and plaques, though the latter association did not remain in multivariable analysis. In a previous study VCAM was associated with arterial stiffness in patients with SSc [44]. We reported recently in a prospective study that VCAM-1 levels predicted cardiovascular mortality in SLE [45]. The present observation adds further support for a pivotal role of the endothelium in autoimmune vascular disease.

Plaques were more common in ACA+ SSc patients, compared both to ACA-patients and controls. This difference remained after adjustment for disease duration, gender and age.

It is of note that, despite the enhanced occurrence of ischemic arterial events and plaques, several traditional CVD risk factors (waist-hip ratio, plasma glucose and triglycerides) had a less 'atherogenic' profile in the ACA+ as compared to the ACA-patients. Another important observation is that ischemic events were rare in the ATA+ group. These patients are part of the ACA-group, which consists of patients with different autoantibody profiles. In previous studies, ACA+ patients had a different disease presentation with more localized skin involvement, calcinosis [46], digital loss [26] and also a different genetic background [47]. Cheng *et al*. previously noted that the carotid wall of patients with SSc was stiffer than that of controls, and in subgroup analysis stiffness was more pronounced in dcSSc than in lcSSc [33]. Together these results imply that the antibody profiles and associated disease subsets contribute to vascular vulnerability in SSc.

To date this is the largest epidemiologically based study investigating both ischemic macrovascular disease and atherosclerosis in patients with SSc and in comparable population-based controls. Nevertheless, we are underpowered for more detailed subgroup analysis and for multivariable analysis of events. The cross-sectional design is a weakness restricting our investigation to survivors of vascular events. Case definitions in the national registries are based on ICD codes and differ from the definitions used in this study. We were thus restricted to compare event rates to our own controls, and could not reliably use the general population/national registries.

## Conclusions

We report increased prevalence of ischemic arterial events, affecting preferentially the heart and peripheral arteries in SSc patients, especially in ACA+ patients, where also atherosclerosis was enhanced. Our results demonstrate the importance to perform detailed clinical descriptive studies, including subgroup analysis of established diagnostic entities. The importance to investigate both the macro- and microvasculature in SSc is underscored. In particular, ACA+ patients should be monitored closely and modifiable cardiovascular risk factors treated at an early stage to avoid ischemic complications such as amputations and myocardial infarctions.

## Abbreviations

ABI: ankle-brachial index; ACA: anticentromere antibodies; ACR: American College of Rheumatology; ANA: antinuclear antibodies; ANOVA: analysis of variance; ATA: antitopoisomerase 1 antibodies; BP: blood pressure; CCA: common carotid artery; CI: confidence interval; CK-MB: creatine kinase: muscle and brain fraction; CVD: cardiovascular disease; dcSSc: diffuse cutaneous systemic sclerosis; ELISA: enzyme-linked immunosorbent assay; EMG: electromyography; EScSG: European Scleroderma Study Group; GFR: glomerular filtration rate; HDL: high-density lipoprotein; HRCT: high-resolution computed tomography: hsCRP: high-sensitivity C reactive protein; ICAM-1: intercellular adhesion molecule 1; ICVD: ischemic cerebrovascular disease; IFL: immunofluorescence; IHD: ischemic heart disease; IL-6: interleukin 6; IMT: intima-media thickness; IPVD: ischemic peripheral vascular disease; lcSSc: limited cutaneous systemic sclerosis; LDL: low-density lipoprotein; MHz: megahertz; MRI: magnetic resonance imaging; OR: odds ratio; PH: pulmonary hypertension; RA: rheumatoid arthritis; SSA/SSB: Sjögren's syndrome antigen A and B; SSc: systemic sclerosis; SLE: systemic lupus erythematosus; TIA: transitory ischemic attacks; VCAM-1: vascular cell adhesion molecule 1; vWf: von Willebrand factor.

## Competing interests

The authors declare that they have no competing interests.

## Authors' contributions

AN designed the study, acquired, analyzed and interpreted the data and drafted the manuscript. KJU acquired and analyzed the data and drafted the manuscript. LB designed the study, acquired the data and drafted the manuscript. SP coordinated the study, acquired the data and drafted the manuscript. AL coordinated and acquired the laboratory data and drafted the manuscript. ES designed the study, analyzed and interpreted the data and drafted the manuscript. All authors reviewed and approved the final manuscript.

## Supplementary Material

Additional file 1 **Supplementary table **Table 1. Specification of macrovascular events in SSc patients and controls Table 2. Characteristics of ACA+ versus ACA-SSc patients. Click here for file
