# Supplementary material for: Ischemic arterial events and atherosclerosis in patients with systemic sclerosis: a population-based case-control study
Source: Arthritis Res Ther. 2013 Aug 14;15(4):R87. doi: 10.1186/ar4267 (PMC3979018; doi:10.1186/ar4267)
Supplement: Additional file 1 — Supplementary table Table 1. Specification of macrovascular events in SSc patients and controls Table 2. Characteristics of ACA+ versus ACA-SSc patients. [file ar4267-S1.DOCX]

Additional file 1:

Table 1. **Specification of macrovascular events in SSc patients and controls**

| **Gender birth year**  **subgroup** | **Type of event** | **Specification of the different events** |
| --- | --- | --- |
| 1. Female 1942  ACA + | IHD | Arrhythmias + pain/dyspnea on exercise test 2002, coronary angiogram normal. |
| 2. Female 1929  ACA + | IHD  IPVD | Coronary bypass operation because of angina 1991 and 1994. 3-vessel disease.  Myocardial infarction 1995,2000  Critical ischemia left leg 2009, bypass, amputation |
| 3. Female 1930  ACA- | IPVD | Occlusion left subclavia 2005, stent |
| 4. Male 1928  ACA- | IHD | Myocardial infarction 1979 and 2005, rescue PCI 2005, no culprit lesion but occlusion in proximal circumflexa and right coronary artery. |
| 5. Female 1925  ACA+ | IPVD | Claudication + ABI 0.8 left leg, duplex 2009 severe stenosis left leg. . |
| 6. Female 1941  ACA+ | IPVD | Claudication + ABI < 0,85 left leg. Low toe-pressure but no further examination done. |
| 7. Female 1925  ACA+ | IPVD | Critical ischemia, bypass, amputation right leg 2009 |
| 8. Male 1928  ACA- (ANA-) | IHD  IPVD | Angina  Occluded popliteal aneurysm operation right leg 2004, left leg 2005 |
| 9. Female 1952  ACA- | IHD | Myocardial infarction 1999, Coronary by-pass 3-vessels |
| 10. Female 1938  ACA+ | IHD  IPVD  ICVD | Myocardial infarction 2004  By-pass right leg 1999  Stroke 2000 |
| 11.Female 1935  ACA+ | IHD | Coronary by-pass and mitral valve replacement 2008. Multiple coronary artery stenosis. Angina |
| 12. Female 1949  ACA+ | IHD | ST-depressions + chest pain in exercise test. Coronary angio normal 2004 |
| 13. Male 1923  ACA- (ANA-) | IHD  ICVD | Myocardial infarction 1991  Stroke 1997 |
| 14. Female 1939  ACA- | IHD | ST-depressions + chest pain in exercise stress test 1995. No coronary angio done. Nitroglycerin effective. |
| 15. Male 1952  ACA- | IHD | Silent myocardial infarction 2006, based on ECG and echocardiographic findings. |
| 16. Male 1941  ACA- | IPVD | Claudication + ABI <0.8. Significant stenosis arteria femoralis superficialis left leg on duplex 2009. |
| 17. Female 1928  ACA + | IPVD | Claudication + ABI <0.8 left leg. Significant stenosis femoralis superficialis left leg, critical ischemia, gangrenous toes 2010 |
| 18. Female 1949  ACA- | IHD | ST-depressions + chest pain in exercise stress test 2004. No coronary angio done. Nitroglycerine effective. |
| 19. Male 1933  ACA+ | ICVD | Stroke 2002 fronto parietal cerebral infarction left side |
| 20. Female 1938  ACA+ | IHD  ICVD | ST-depressions + chest pain on exercise stress test 2007. No coronary angio done.  Amaurosis fugax 2005 |
| 21. Female 1950  control | IHD | Myocardial infarction 2002, angiography showed subtotal stenosis. No surgical invention |
| 22. Female 1938  control | ICVD | Stroke 2006 |
| 23. Female 1928  control | IHD  ICVD | ST-depressions + chest pain on exercise test. Coronary angiography normal 2008  Stroke 2004 |
| 24. Female 1935  control | ICVD | Stroke 2005 |
| 25. Female 1925  control | IHD | Myocardial infarction, coronary bypass 1998 |
| 26. Female 1944  control | IHD | Silent myocardial infarction 2006 based on syncope and inferior T-wave abnormalities. |
| 27. Male 1923  control | IPVD | Claudication + ABI right leg 0.6, no further investigation performed. |

ACA: anticentromere antibodies, IHD: ischemic heart disease, IPVD: ischemic peripheral vascular disease, ICVD: ischemic cerebral vascular disease.

Table 2. **Characteristics of ACA+ versus ACA- SSc patients**

|  | **ACA +**  **(n=34)** | **ACA –**  **(n=77)** | **p-value** |
| --- | --- | --- | --- |
| Age years | 64.1 ± 11.8 | 60.9 ± 12.7 | ns |
| Gender female | 92% | 76% | ns (0.07) |
| **Disease Characteristics** |  |  |  |
| Disease duration | 11.4 (7.8-18.5) | 8.5(4.6-16.3) | 0.04 |
| Diffuse cutaneous  Limited cutaneous | 6%  94% | 29%  71% | 0.03 |
| **Autoantibodies**  ACA  ATA  ANA-negative | 100%  6%  0% | 0%  31%  11 % | 0.003 |
| Pulmonary fibrosis | 18% | 58% | 0.0001 |
| Pulmonary hypertension | 18% | 16% | ns |
| Digital Ulcers ever | 41 % | 36 % | ns |
| Calcinosis | 35% | 22% | ns |
| Myositis | 6% | 10% | ns |
| Kidney involvement | 3% | 9% | ns |
| Skin score | 6 (4-11) | 6 (3-10) | ns |
| Arthritis | 35% | 27% | ns |
| Disease activity | 0.5 (0.12 – 1.87) | 0.5 (0 – 2.0) | ns |
| Disease damage | 5 (2.25 – 6) | 5 (3 – 6) | ns |
| **Traditional risk factors** |  |  |  |
| Body Mass Index | 23.98 ± 2.60 | 24.32 ± 4.23 | ns |
| Waist hip ratio | 0.80 ± 0.07 | 0.85 ± 0.10 | 0.008 |
| Ever smoked %  Current smoker % | 53 %  8 % | 53 %  13 % | ns  ns |
| Systolic BP (mmHg)  Diastolic BP (mmHg)  Hypertension % | 124.92± 18.11  71.45 ± 11.8  19 % | 122.4 ± 17.9  71.70 ± 10.2  30 % | ns  ns  ns |
| P-glucose (mmol/l) | 5.0 (4.7 – 5.3) | 5.2 (4.9 – 5.7) | 0.04 |
| Diabetes %  Cholesterol (mmol/l)  LDL (mmol/l)  HDL (mmol/l)  Triglycerides (mmol/l) | 0 %  5.36 ± 1.13  3.44 ± 0.91  1.4 (1.07 – 1.7)  0.88 (0.68 - 1.22) | 9 %  5.13 ± 1.00  3.27 ± 0.95  1.3 (1 -1.6)  1.0 (0.79 – 1.6) | ns (0.06)  ns  ns  ns  0.05 |
| **Inflammatory biomarkers** |  |  |  |
| hsCRP (mg/l) | 1.9(0.9 – 3.6) | 2.7 (1 – 4.9) | ns |
| Orosomucoid (g/l) | 0.8 (0.7 – 0.9) | 0.8 (0.7 – 1.0) | 0.05 |
| Alpha1antitrypsine (g/l) | 1.5 (1.4 – 1.6) | 1.5 (1.3 – 1.6) | ns (0.07) |
| Fibrinogen (g/l) | 3.7 (3.2– 4.1) | 3.7(3.3 – 4.6) | ns |
| Sedimentation Rate (mm) | 12.5 (9 – 18) | 16 (8– 29) | ns |
| IL-6 (pg/ml) | 27.1(20.0 – 39.1) | 31.4 (23.9-41.0) | ns |
| **Endothelial biomarkers** |  |  |  |
| VCAM-1 (ng/L) | 730 (565 – 872) | 627 (508 – 790) | ns |
| ICAM-1 (ng/L) | 347(295 – 423) | 405 (325 – 494) | 0.01 |
| VEGF (pg/ml) | 177.5 (134.2-298.0) | 184.0 (147.3-244.7) | ns |
| vWF (UI/ml) | 1.26 (0.8 – 1.6) | 1.21 (0.9 – 1.8) | ns |
| **Kidney function test**  eGFR (mL/min/1.73 m^2^) | 90 (78 – 104) | 93 (73 – 116) | ns |

Distributions are given as %, median (interquartile range) or mean ± standard deviation.

ACA: anticentromere antibody, LDL: low density lipoprotein, HDL: high density lipoprotein, IL: interleukin,VCAM: vascular cell adhesion molecule, ICAM: intercellular adhesions molecule, VEGF: vascular endothelial growth factor, vWF: von Willebrand factor, eGFR: estimated glomerular filtration rate, based on Cystatin C measurements (15)
